# Leveraging multimodal neuroimaging and GWAS for identifying modality-level causal pathways to Alzheimer’s disease

**DOI:** 10.1162/imag_a_00580

**Published:** 2025-05-16

**Authors:** Yuan Tian, Daniel Felsky, Jessica Gronsbell, Jun Young Park

**Affiliations:** Department of Statistical Sciences, University of Toronto, Toronto, ON, Canada; Krembil Centre for Neuroinformatics, Centre for Addiction and Mental Health, Toronto, ON, Canada; Division of Biostatistics, Dalla Lana School of Public Health, University of Toronto, Toronto, ON, Canada; Institute of Medical Science, University of Toronto, Toronto, ON, Canada; Department of Psychiatry, University of Toronto, Toronto, ON, Canada; Department of Computer Science, University of Toronto, Toronto, ON, Canada; Department of Family & Community Medicine, University of Toronto, Toronto, ON, Canada; Department of Psychology, University of Toronto, Toronto, ON, Canada

**Keywords:** Alzheimer’s disease, causal inference, instrumental variable, imaging-derived phenotypes, summary statistics, TWAS

## Abstract

The UK Biobank study has produced thousands of brain imaging-derived phenotypes (IDPs) collected from more than 40,000 genotyped individuals so far, facilitating the investigation of genetic and imaging biomarkers for brain disorders. Motivated by efforts in genetics to integrate gene expression levels with genome-wide association studies (GWASs), recent methods in imaging genetics adopted an instrumental variable (IV) approach to identify causal IDPs for brain disorders. However, several methodological challenges arise with existing methods in achieving causality in imaging genetics, including horizontal pleiotropy and high dimensionality of candidate IVs. In this work, we propose testing the causality of each brain modality (i.e., structural, functional, and diffusion magnetic resonance imaging (MRI)) for each gene as a useful alternative, which offers an enhanced understanding of the roles of genetic variants and imaging features on behavior by controlling for the pleiotropic effects of IDPs from other imaging modalities. We demonstrate the utility of the proposed method by using Alzheimer’s GWAS data from the UK Biobank and the International Genomics of Alzheimer’s Project (IGAP) study. Our method is implemented using summary statistics, which is available on GitHub.

## 1 Introduction

Studies indicate that dementia, including Alzheimer’s disease (AD), is highly heritable, and genetic factors are estimated to play a role in around 80% of AD cases (Gatz et al., 2006;Tanzi, 2012). However, research on dementia still has significant room for developing genetics–brain pathways. Although genome-wide association studies (GWASs) have identified a number of genetic risk factors for AD, they are limited in testing for a direct association between the variants and the disease trait. GWASs do not provide a comprehensive understanding of how the genetically regulated structural and functional brain pathways drive AD progression, which is essential for characterizing the genetic mechanism of the disease.

The growing interest in the role of genetics in brain disorders has spawned a new field known as imaging genetics that integrates neuroimaging features (e.g., from brain magnetic resonance imaging (MRI)) with genetics using statistics and machine learning. Many neuroimaging features, including the brain’s anatomy (structural MRI (sMRI)), function (functional MRI (fMRI)), and microstructure (diffusion MRI (dMRI)), have been demonstrated to be heritable. For example,Lin et al. (2014)andHuang et al. (2015)use imaging features as GWASs traits to identify the associations between the imaging features and single nucleotide polymorphisms (SNPs) or a set of SNPs (e.g., a gene). At the same time, in recent years, there has been substantial evidence in the studies of brain disorders that brain MRI features, that is, imaging-derived phenotypes (IDPs), are useful biomarkers for AD and are modulated by genetic factors (Elliott et al., 2018). By integrating data from imaging scans and GWASs, researchers have attempted to better understand the heritability of AD and other brain-related diseases. Such integrative analyses have been promising in advancing imaging neuroscience that paces with an increased size of genetic and neuroimaging data (Shen & Thompson, 2019). However, due to its high dimensionality (e.g., thousands of IDPs available from UK Biobank (Elliott et al., 2018;Smith et al., 2021)) and complex correlation structure (e.g., correlations between imaging features), it is difficult to fully leverage brain imaging data in GWASs, especially in pinpointing causal imaging features affecting the heritability of brain-related diseases (Burgess et al., 2017).

The transcriptome-wide association study (TWAS), which leverages expression quantitative trait loci (eQTL) with GWAS, has become an important statistical tool in genetics research (Gamazon et al., 2015). TWAS identifies genes associated with diseases/phenotypes through genetic regulation of gene expression. TWAS is constructed by first using SNPs in a gene to predict the corresponding gene expression levels via machine learning methods (e.g., penalized regression), which represents the “genetically regulated” (or “genetically imputed”/“genetically predicted”) component of gene expression. The predicted gene expression levels are then associated with selected outcomes, adjusting for multiple comparisons. In neuroimaging literature, the imaging-wide association study (IWAS) extended TWAS by considering multiple IDPs as intermediate phenotypes rather than gene expression, which may offer improved mechanistic interpretation when studying brain disorders such as AD (Xu et al., 2017). These approaches showed significant improvement in localizing genes associated with the phenotype compared with GWAS.

In addition, TWAS offers a statistical interpretation from the lens of causal inference via instrumental variable analysis. Specifically, TWAS is equivalent to testing a causal relationship from eQTL to phenotype using 2-stage least squares (2SLS) by using SNPs as instrumental variables (Mai et al., 2023;Xue et al., 2020). In neuroimaging, BrainXcan takes this approach to identify IDPs leading psychiatric traits under the assumptions of Mendelian Randomization (MR) (Liang et al., 2025). The multivariate IWAS (MV-IWAS) extends the causal interpretation of TWAS in imaging genetics by accounting for horizontal pleiotropic effects of other IDPs (Knutson et al., 2020). We refer toTaschler et al. (2022)for a comprehensive review of conducting causal inference with neuroimaging data using MR. A key consideration in these approaches in imaging genetics is the high dimensionality of the IDPs. For example, the UK Biobank provides thousands of IDPs, which may increase dramatically based on preprocessing pipelines and analysis goals (e.g., ROI-level analysis versus whole-brain analysis). Because it increases the burden of multiple comparisons, BrainXcan and MV-IWAS consider the whole genome as potential instrumental variables and use polygenic scores (PGSs) to implement the first stage of 2SLS. However, such approaches would lose the original purpose of TWAS, which was to localize gene-level associations with diseases/phenotypes.

This paper aims to mitigate current challenges in making causal interpretations in imaging genetics and localizing corresponding genes. Rather than evaluating each IDP individually, we propose to make causal interpretations between diseases/phenotypes and each*imaging modality*(e.g., sMRI, fMRI, or dMRI). Each modality represents distinct aspects of brain structure and function, and therefore, aggregating the brain information in each modality can contribute to a more comprehensive understanding of the genetic mechanisms underlying brain disorders. At the same time, our method explicitly accounts for horizontal pleiotropy among imaging modalities, therefore, providing robustness of the causal direction between each modality and the progression of AD. With the MRI modalities through which genetics affect brain function in AD, we can better understand the underlying biological mechanism by which genetics causes changes in brain function, which in turn causes AD. We also make our methods implementable using GWAS summary statistics and reference panels, enhancing the practical utility.

The rest of the paper is organized as follows. InSection 2, we provide a methodological review of the statistical methods in imaging genetics that extended TWAS and develop the proposed method, including the implementation with summary statistics. InSection 3, we evaluate Type 1 error rate and power of the proposed method by simulations.Section 4applies our methods to the AD GWAS data obtained by the UK Biobank and the International Genomics of Alzheimer’s Project (IGAP). A few points of discussion are made inSection 5.

## 2 Methods

### 2.1 Notations

We let$\text{y}=(y_{1},\ldots ,y_{N}{)}^{\prime }$denote the phenotype of our interest (e.g., AD status) fromNsubjects. Also, let${\text{g}}_{j}=(g_{1j},\ldots ,g_{Nj}{)}^{\prime }$be the collection of thejth SNP across subjects in a gene forj=1,…,J. Similarly, we let${\text{m}}_{k}\;=(m_{1k},\ldots ,m_{Nk}{)}^{\prime }$be thekth IDP fork=1,…,K. Without loss of generality, we assume that each${\text{g}}_{j}$and${\text{m}}_{k}$is standardized to have the zero mean and unit variance.

### 2.2 Review of existing methods

We first review transcriptome-wide association study (TWAS) and relevant approaches in neuroimaging.

#### 2.2.1 TWAS and UV-IWAS

With a single endophenotypem, TWAS is a method that integrates endophenotypes (e.g., gene expression) in gene-based association study to test the genetically regulated effect of the endophenotype on the disease trait (Gusev et al., 2016). TWAS is conducted using a two-stage approach. In Stage 1, using a set of SNPs encoded in a gene$\left({\text{g}}_{1},\ldots ,{\text{g}}_{J}\right)$, machine learning methods (e.g., linear models) are used to predict a corresponding gene expression level (m) based on the following model:

$$\text{(TWAS)}\;\text{stage }1:\text{E}[\text{m}]=\sum_{j=1}^{J}{\text{g}}_{j}\alpha_{j}.$$

Here,$\left({\hat{\alpha }}_{1},\ldots ,{\hat{\alpha }}_{J}\right)$is obtained by fitting a penalized linear regression with the ridge (Liang et al., 2025), LASSO, or elastic net penalty (Gusev et al., 2016). Then, a*genetically imputed*expression,$\hat{\text{m}}=\sum_{j=1}^{J}{\text{g}}_{j}{\hat{\alpha }}_{j}$, is used to test for its association with the phenotype (y) in Stage 2 working model:

$$\text{(TWAS) stage 2: }h\left(\text{E}[\text{y}]\right)=\hat{m}\;\beta ,$$

whereh(⋅)is the canonical link function (e.g., the logistic link for binary traits and the identity link for for quantitative traits). TWAS tests$H_{0}:\beta =0$to identify genes whose genetically regulated gene expression is associated with the trait. UV-IWAS is conceptually equivalent to TWAS (Knutson et al., 2020), using an imaging-derived phenotype (IDP) instead for gene expression as an endophenotype. One insight of UV-IWAS is that AD is primarily a brain disorder, and using IDPs may be more effective than gene expression in AD studies.

TWAS (or UV-IWAS) provides a useful framework for understanding how genetic variation influences disease by modeling intermediate endophenotypes, such as gene expression or IDPs, which help capture key aspects of biological function at the transcriptomic or neuroanatomical level. It also offers several possible statistical interpretations. One interpretation is that it is essentially a weighted burden test for gene-based association testing where the weight for the j th SNP in a gene is determined by ${\hat{\alpha }}_{j}$ in Stage 1. Because an optimal choice of weights would lead to improved power in the burden test, the choice of weights in TWAS, which is informed biologically, is expected to improve statistical power. Another interpretation is that it can be interpreted as an instrumental variable (IV) approach, where the 2-stage least squares (2SLS) is used to estimate and infer the *causal* effect of endophenotypes ( m ) on the phenotype ( y ). In such a case, the SNPs that led the causal direction serve as composite IVs, provided that the following assumptions hold.

A1.  There exist correlations between all SNPs selected in Stage 1 and the endophenotype;
A2.  SNPs are uncorrelated with potential confounders;
A3.  SNPs do not affect the disease trait, except through the endophenotype/IDP being tested.

A3 indicates that the SNPs do not affect the disease trait via genetic direct effect. Since most SNPs are unrelated to endophenotypes, certain thresholds are typically used in TWAS Stage 1, such as predictive$R^{2}$orpvalue, to ensure that A1 is met.

#### 2.2.2 Multivariate IWAS (MV-IWAS)

A unique challenge in imaging genetics is that there are multiple IDPs each may have distinct genetic regulatory pathways and potential pleiotropic effects to AD. However, multiple significant IDPs have been identified (Xu et al., 2017); as discussed byKnutson et al. (2020)andElliott et al. (2018), the presence of horizontal pleiotropy can lead to violations of the IV assumptions, resulting in invalid causal inference using univariate IV analysis and ultimately inflating Type I error in the hypothesis testing for causal effects from an IDP to a phenotype, especially when there is high correlation between the IDPs.

To address the potentially inflated Type I error rate due to pleiotropy,Knutson et al. (2020)proposed using multiple linear regression in Stage 2 to adjust for possible pleiotropic effects of genetically regulated IDPs from gene to disease. If there areKimaging features in total, MV-IWAS considers the working model the second stage:

$$\begin{array}{l} \text{(MV-IWAS) stage 1: E}[{\text{m}}_{k}]=\sum_{j=1}^{J}\;{\text{g}}_{j}\alpha_{jk},\\ \text{(MV-IWAS) stage 2: }h\left(\text{E}[\text{y}]\right)=\sum_{k=1}^{K}{\hat{\text{m}}}_{k}\beta_{k}. \end{array}$$

Stage 1 of MV-IWAS is equivalent to Stage 1 of TWAS/UV-IWAS, where each IDP is predicted separately by the same set of SNPs across all chromosomes. In Stage 2, a general linear model (GLM) is applied using all predicted endophenotypes, replacing the univariate regression in TWAS/UV-IWAS. In MV-IWAS, whether thekth IDP causes the phenotype is achieved by testing$H_{0k}:\beta_{k}\;=0$in the Stage 2 model, which controls the pleiotropic effects of the other IDPs. Furthermore, to account for remaining genetic direct effects,Knutson et al. (2020)extended the Stage 2 model to MV-IWAS-Egger to account for direct genetic effects on the phenotype. The Stage 2 model of MV-IWAS-Egger is formulated by

$$\text{(MV-IWAS-Egger) stage 2: }h\left(\text{E}[\text{y}]\right)=\left(\sum_{j=1}^{J}\;{\text{g}}_{j}\right)\cdot \mu +\sum_{k=1}^{K}{\hat{\text{m}}}_{k}\beta_{k},$$

with the same null hypothesis$H_{0k}:\beta_{k}=0$to test the causal effect of thekth IDP on the phenotype. We note that, from its construction, the power of MV-IWAS and MV-IWAS-Egger is severely affected by the multicollinearity of the${\hat{\text{m}}}_{k}$s (and$\sum_{j=1}^{J}{\text{g}}_{j}$for MV-IWAS-Egger) (Burgess et al., 2017).

#### 2.2.3 Remaining challenges

While MV-IWAS provides a possible causal interpretation, it could be underpowered due to the following factors:

1.*Multicollinearity*: Multicollinearity arises when two genetically regulated components of the IDPs (e.g.,${\hat{\text{m}}}_{1}$and${\hat{\text{m}}}_{2}$) are highly correlated, leading to a challenge in point estimation (i.e., an increase in the standard error) in Stage 2 of MV-IWAS and a decrease in statistical power. We refer toChan et al. (2024)for the challenges with applying Mendelian randomization in case of high dimensional exposures (IDPs).

We empirically evaluated the multicollinearity by computing the genetic correlations of the IDPs. Specifically, we performed Linkage Disequilibrium Score Regression (LDSC) analyses (Duncan et al., 2018) using UKB IDP GWAS summary statistics available fromSmith et al. (2021). For computational reasons, we included top 50 features with highest heritability estimates from different imaging modalities. As shown inFigure 1, 28.5% of sMRI and 60.3% of dMRI within modality IDP pairs show genetic correlation greater than0.7, which implies that including such highly correlated IDPs in MV-IWAS could suffer from power loss.

**Fig. 1.**
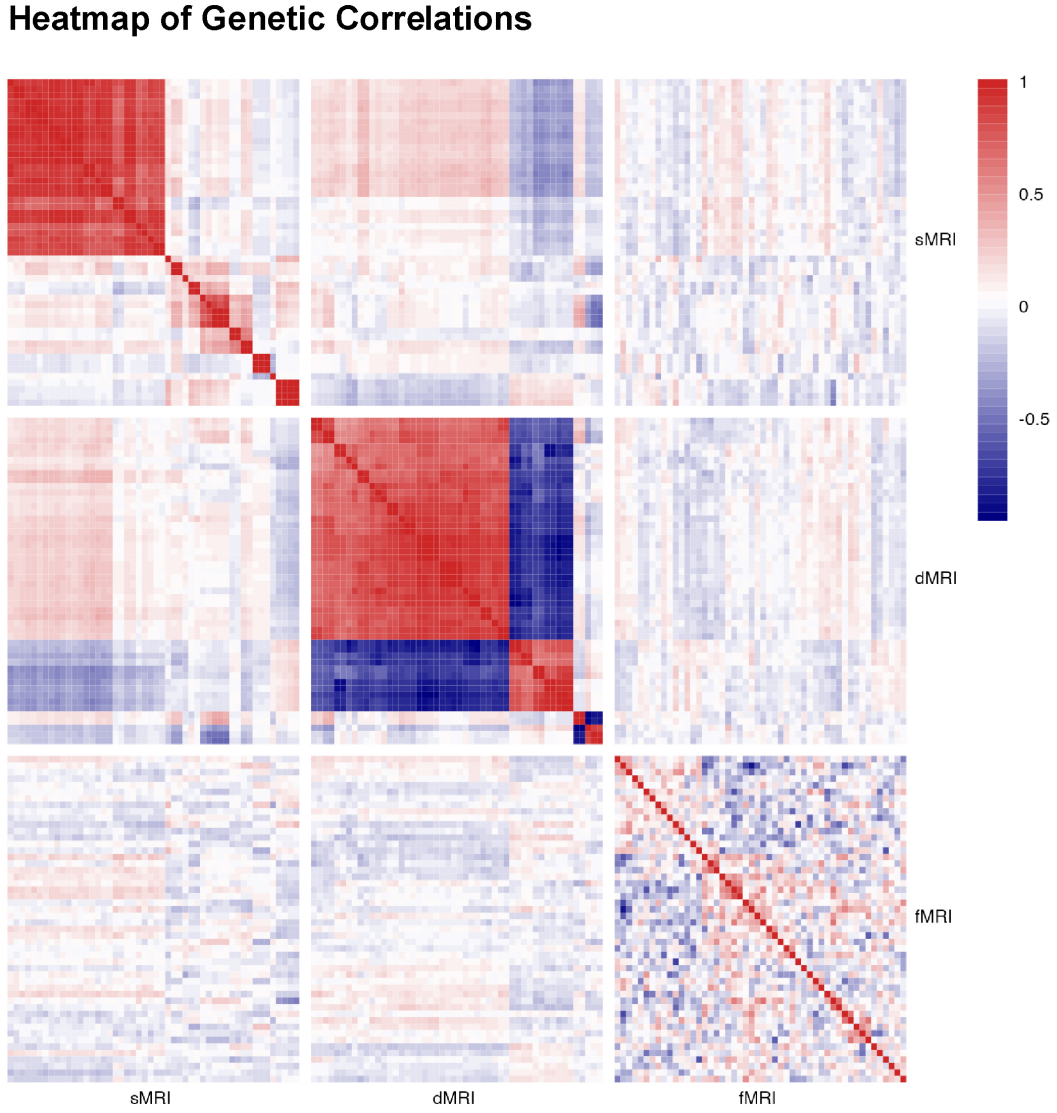
Genetic correlations among the top 50 heritable IDPs (according toSmith et al. (2021))from structural MRI (sMRI), diffusion MRI (dMRI), and functional MRI (fMRI). The correlations were estimated using LDSC, using all SNPs across all chromosomes with summary statistics from UKB IDP GWAS and reference panel from 1000G, with a minor allele frequency (MAF) filter of 1%. IDPs within each modality were reordered by hierarchical clustering for better visualization.

2.*Multiple testing*: Multiple testing becomes an issue when multiple highly correlated IDPs are associated with a gene, increasing the number of comparisons in Stage 2 and making a stringent Bonferroni correction necessary, which can substantially reduce statistical power.

### 2.3 Proposed method: testing modality-specific pathways to AD

To mitigate potential power loss in analyzing multiple IDPs in an IV framework, we propose testing all IDPs in each MRI modality as a unitary entity while controlling for pleiotropic effects from other modalities. Referring back toFigure 1, we largely observed high genetic correlations within each MRI modality, including such high correlated IPDs in MV-IWAS can compromise inference accuracy, which motivated us to test all IDPs at the modality level. Besides the high within-modality correlations, we also noted moderate cross-modality correlations, for example, between sMRI and dMRI IDPs (Fig. 1), indicating a need to adjust for cross-modality pleiotropic effects. Beyond statistical considerations, grouping IDPs by modality is also scientifically informative: each MRI modality characterizes distinct aspects of brain structure and function, and, therefore, aggregating the brain information in each modality can contribute to a more comprehensive understanding of the genetic mechanisms underlying neurological diseases.

To the end, we specifically aim to answer the question:*how does a gene lead to AD progression through which imaging modality*? To handle possible bi-directional genetic effects, for example, bidirectional effect of dMRI IDPs inFigure 1, we propose MV-Modality-IWAS (multivariate modality global testing in IWAS), which bridges the extremes of testing each IDP individually in MV-IWAS (Knutson et al., 2020) and testing all IDPs together through a global testing in IWAS (Xu et al., 2017). By testing all IDPS within each MRI modality, we can assess the genetic regulatory effects of entire sets of imaging features, rather than individual IDPs, which may capture a range of subtle and complex brain processes. Ultimately, this methodology has the potential to uncover novel genetic variants or regions linked to brain structure and function, providing new insights into the underlying mechanisms of AD. A graphical illustration is provided inFigure 2.

**Fig. 2.**
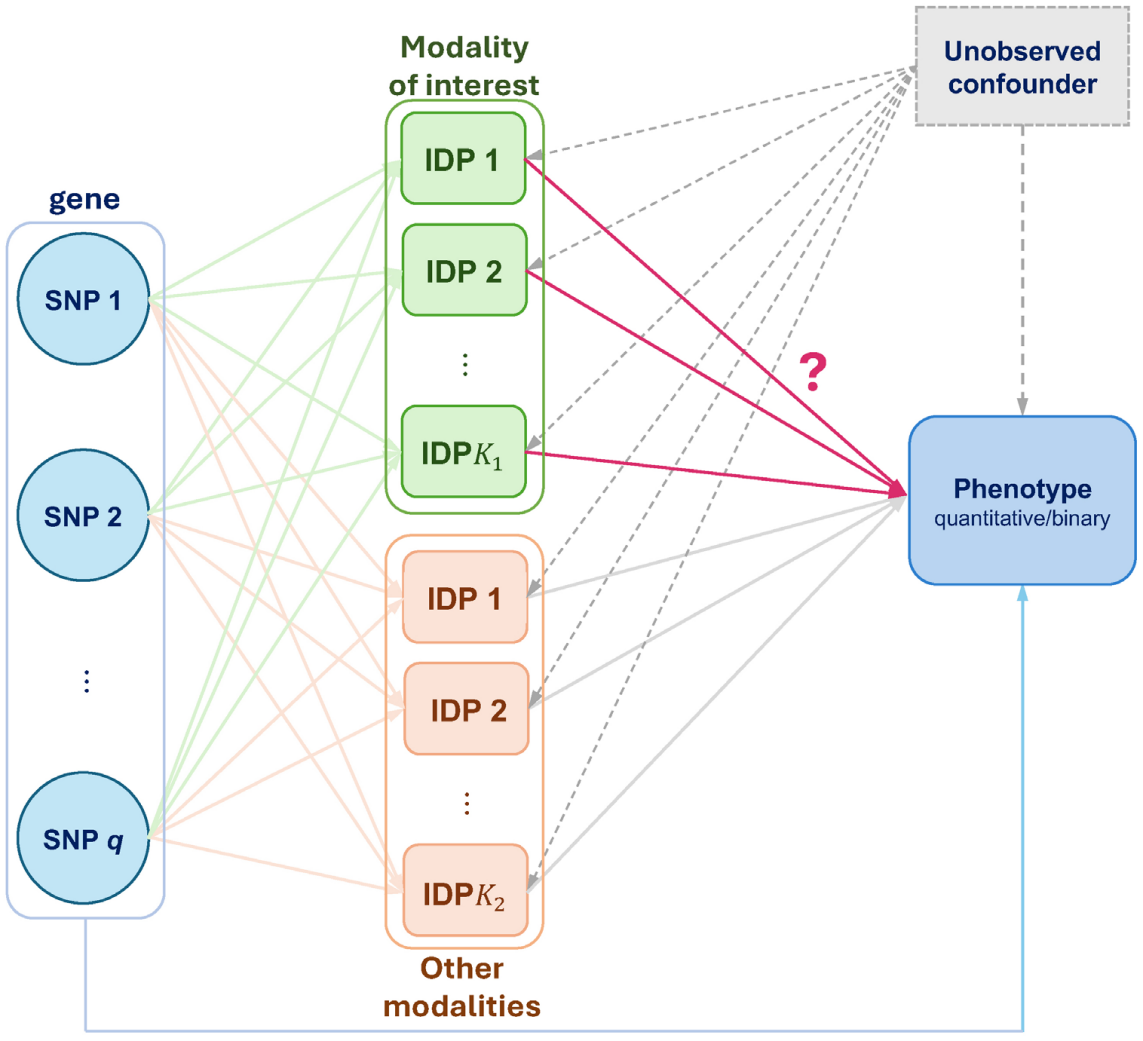
An illustration of a data-generating model for simulation studies, using a directed acyclic graph (DAG) representation. Different colors in IDP denote different imaging modalities, where the green one is the modality of our interest.

Our Stage 2 model considers the testing$H_{0}:\beta_{1}^{\left(1\right)}=\cdots$$=\beta_{K_{1}}^{\left(1\right)}=0$for each gene from the following working model:

$$h\left(\text{E}[\text{y}]\right)=\left(\sum_{j=1}^{J}{\text{g}}_{j}\right)\cdot \mu +\sum_{k=1}^{K_{1}}{\hat{\text{m}}}_{k}^{\left(1\right)}\beta_{k}^{\left(1\right)}+\sum_{k=1}^{K_{2}}{\hat{\text{m}}}_{k}^{\left(2\right)}\beta_{k}^{\left(2\right)}.$$ (1)

Equation (1)provides an interpretable decomposition of genetic pathways to phenotype: (i) the direct effect (adjusted byμ) and (ii) genetically regulated effects (modeled by$\beta_{1}^{\left(q\right)},\ldots ,\beta_{K_{q}}^{\left(q\right)}$) through theqth imaging modality. With the assumed model, we test whether the specified modality contributes to the phenotypey, that is, test the existence of casual modality-specific genetic pathways.

We propose to test the genetically regulated effects of a given modality using score-based testing while adjusting genetically regulated effects from other modalities, as well as residual genetic direct effects, as fixed effects, with the test statisticT,

$$T=\sum_{k=1}^{K_{1}}{\left(\frac{1}{N}\cdot {\hat{\text{m}}}_{k}^{(1{)}^{\prime }}\left(\text{y}-{\tilde{\text{y}}}^{\left(1\right)}\right)\right)}^{2}$$ (2)

where${\tilde{\text{y}}}^{\left(1\right)}=h^{-1}\left(\left(\sum_{j=1}^{J}g_{j}\right)\tilde{\mu }+\sum_{k=1}^{K_{2}}{\hat{\text{m}}}_{k}^{\left(2\right)}\;{\tilde{\beta }}_{k}^{\left(2\right)}\right)$,$\tilde{\mu }$and${\tilde{\beta }}_{k}^{\left(2\right)}$s are estimated under the null hypothesis. Under the null hypothesis,Tasymptotically follows a mixture$\chi_{1}^{2}$distribution, which is approximated by a single non-central chi-square distribution with matching moments (Davies, 1980). In the proposed modality-level testing, the fixed effects of genetically regulated effects from other modalities are adjusted, similarly to the format of the Sequence Kernel Association Test (SKAT) (Wu et al., 2011). The proposed modality-level testing is motivated here well because (i) each IDP contributes only a small proportion to the progression of AD and (ii) the directions (sign) of the effects are expected to be non-uniform. In this scenario, different IDPs may drive AD in varying effect directions and magnitudes, in which SKAT is known to be powerful in this context. We note, however, that other test statistics could also be considered, such as score-based tests (Pan et al., 2014) (when accounting for sparsity inβ), orF-type tests (Knutson et al., 2020) (under the normality assumption fory).

The proposed test is an extension of several previous methods including UV-IWAS and multivariate model in MV-IWAS. If only one IDP in the modality being tested is included, it is equivalent to UV-IWAS. However, the proposed test differs from UV-IWAS in that it adjusts for other modalities, which could lead to horizontal pleiotropy. If the modality has one feature only, the proposed test is also equivalent to MV-IWAS or MV-IWAS-Egger. Our proposed method can be viewed as an extension of previous methods but can handle the challenges of testing genetically regulated effects of imaging features in the modality of interest while adjusting for genetic direct effects and effects from other modalities.

### 2.4 Implementation using GWAS summary statistics

Our method can be implemented using reference GWAS summary statistics, making it more useful in practice. For simplicity in notations, we define$\text{G}=[{\text{g}}_{1},\ldots ,{\text{g}}_{J}]$and let${\hat{\text{A}}}^{\left(1\right)}\;=\;\;{\left[{\hat{\alpha }}_{jk}^{\left(1\right)}\right]}_{j=1,k=1}^{J,K_{1}}$be a coefficient matrix for the modality (of the interest) from the Stage 1 working model forj=1,…,Jand$k=1,\ldots ,K_{1}$. We define${\hat{\text{A}}}^{\left(2\right)}$for all other modalities, including direct effects.

Then the Stage 2 model is summarized by testing$H_{0}:\beta^{\left(1\right)}={\left(\beta_{1}^{\left(1\right)},\ldots ,\beta_{K_{1}}^{\left(1\right)}\right)}^{\prime }=0$in

$$\text{y}=\text{G}{\hat{\text{A}}}^{\left(1\right)}\beta^{\left(1\right)}+\text{G}{\hat{\text{A}}}^{\left(2\right)}\beta^{\left(2\right)}+\epsilon ,$$

and the test statistic inEquation (2)is equivalent to

$$\underset{{\text{S}}^{\prime }}{\underset{︸}{T=\frac{1}{N}{\left(\text{y}-\text{G}{\hat{\text{A}}}^{\left(2\right)}{\tilde{\beta }}^{\left(2\right)}\right)}^{\prime }\text{G}{\hat{\text{A}}}^{\left(1\right)}}}\cdot \underset{\text{S}}{\underset{︸}{\frac{1}{N}{\hat{\text{A}}}^{(1{)}^{\prime }}{\text{G}}^{\prime }\left(\text{y}-\text{G}{\hat{\text{A}}}^{\left(2\right)}{\tilde{\beta }}^{\left(2\right)}\right)}}.$$ (3)

Here,

S=1N⋅A^(1)′G′(y−GA^(2)β˜(2)) =1NA^(1)′G′y−1NA(1)′G′GA^(2)(A^(2)′G′GA^(2))−1A^(2)′G′y ≈A^(1)′z−A^(1)′RA^(2)(A^(2)′RA^(2))−1A^(2)′z,

where$\text{z}=(z_{1},\ldots ,z_{J})={\text{G}}^{\prime }\text{y}/(N-1)$is the vector of z-scores of the GWAS between the phenotype and a gene, and$\text{R}={\text{G}}^{\prime }\text{G}\;/(N-1)$is the pairwise Pearson correlation matrix of SNPs in the gene, followingKwak and Pan (2016). Note thatRcould also be approximated by a reference panel (e.g., 1000 Genomes Project (The 1000 Genomes Project Consortium, 2010)). Similarly, we get

$$\hat{Cov}(\text{S})\approx {\hat{\text{A}}}^{(1{)}^{\prime }}\text{R}{\hat{\text{A}}}^{\left(1\right)}-{\hat{\text{A}}}^{(1{)}^{\prime }}\text{R}{\hat{\text{A}}}^{\left(2\right)}{\left({\hat{\text{A}}}^{(2{)}^{\prime }}\text{R}{\hat{\text{A}}}^{\left(2\right)}\right)}^{-1}{\hat{\text{A}}}^{(2{)}^{\prime }}\text{R}{\hat{\text{A}}}^{\left(1\right)}.$$

SinceSfollows a multivariate normal distribution with the zero mean under the null hypothesis, it is sufficient to use plug-in estimate$\hat{Cov}(\text{S})$to derive the null distribution ofT(mixture$\chi_{1}^{2}$). We use the CompQuadForm R package to obtain thepvalue when$K_{1}>5$and use the Monte Carlo method to obtain thepvalue otherwise to improve suboptimal Davies approximation methods to the mixture$\chi_{1}^{2}$distribution of the test statistic.

We note that our summary statistics approach implicitly assumes that the phenotype of interest is continuous, which is violated when working with binary disease status. However, such “linearization” is a well-accepted method when the effect sizes of the covariates are tiny, which is also the case when evaluating the effects of imputed IDPs to AD (Knutson et al., 2020;Xue et al., 2020). To evaluate the robustness, our simulation studies inSection 3are implemented by the summary statistics approach in this section, while the logistic model is used. The R functions to implement the proposed summary statistics approach are available athttps://github.com/junjypark/MV_Modality_IWAS.

## 3 Simulation Studies

### 3.1 Simulation designs

We conducted simulation studies to evaluate Type 1 error rates and statistical power under linear model assumptions in both Stages 1 and 2, extending simulations ofKnutson et al. (2020). We generatedn=2,000subjects and usedJ=58SNPs with their correlation structures obtained from a gene on chromosome 16. The SNP data were generated by using the rmvbin function from the bindata R package.

Two imaging modalities were generated (q=1,2), each with 10 features. A graphical model inFigure 2was used to generate simulated imaging features and binary phenotypes. In our simulations, all SNPs were assumed to be associated with all imaging features. There is an unobserved confounding variableuwhich is used to generate imaging features and the phenotype (but not used to computepvalues). Therefore, the imaging features were generated by

$${\text{m}}_{k}^{\left(q\right)}=\sum_{j=1}^{J}\;{\text{g}}_{j}\alpha_{jk}^{\left(q\right)}+\text{u}\gamma_{k}^{\left(q\right)}+\delta_{k}^{\left(q\right)},$$

where$\delta_{k}^{\left(q\right)}$is the residual noise. The binary phenotype was generated via (i) effects from imaging features, (ii) direct effects from genotypes, and (iii) effects from the unobserved confounding variable. Specifically,

$$\begin{array}{l} \text{(Continuous trait)}\;\text{y}=\left(\sum_{j=1}^{J}\;{\text{g}}_{j}\right)\cdot \mu +\sum_{q=1}^{2}\sum_{k=1}^{K}\;{\text{m}}_{k}^{\left(q\right)}\beta_{k}^{\left(q\right)}+\text{u}\cdot \theta +\epsilon ,\\ \;\;\text{(Binary trait)}\;\text{logit}\left(\text{E}(\text{y})\right)=-2+\left(\sum_{j=1}^{J}\;{\text{g}}_{j}\right)\cdot \mu +\sum_{q=1}^{2}\sum_{k=1}^{K}\;{\text{m}}_{k}^{\left(q\right)}\beta_{k}^{\left(q\right)}+\text{u}\cdot \theta . \end{array}$$

The parameters in our simulation studies were chosen to reflect gene-level effects on imaging features and phenotypes. Specifically, we used$\alpha_{jk}^{\left(q\right)}∼N(0,0.3^{2})$,$\gamma_{k}^{\left(q\right)}∼N(0,1^{2})$,$\beta_{k}^{\left(2\right)}∼N(0,0.05^{2})$,μ=1, andθ=1. The variance of noise of the IDPs ($\delta_{k}^{\left(q\right)}$) and the continuous trait were set to be$5^{2}$and$4^{2}$, respectively. In both continuous and binary traits, the average$R^{2}$for each of (i) genotype–phenotype and (ii) genotype–IDP was set to be no larger than 0.10 on average under the null model. The average$R^{2}$for IDP–phenotype was also set to be no larger than 0.10 in the continuous trait setting. Also, in binary traits, the prevalence of AD was approximately 18%.

For the power analysis, we consider two different scenarios. In**Scenario 1**, allq=10IDPs in the first imaging modality cause the trait and$\beta_{1}^{\left(1\right)},\ldots ,\beta_{q}^{\left(1\right)}∼N(0,\tau^{2}),$whereτcontrols the degree of signals. In**Scenario 2**, only the first IDP was causal and the remaining IDPs were not (i.e.,$\beta_{2}^{\left(1\right)}=\cdots =\beta_{q}^{\left(1\right)}=0)$.

Denoting our method as**Method 1 (Proposed)**, we compare our methods with three competitors.**Method 2 (IDP-specific)**is the univariate version of the proposed method, where the pleiotropic effect of the other modalities is adjusted but each feature of the modality of interest is tested separately. The global Type 1 error is controlled by applying Bonferroni correction (i.e.,α​/​10for 10 features in the modality).**Method 3 (Proposed unadjusted)**is our method without adjusting for the pleiotropic effect of the other modalities.**Method 4 (UV-IWAS)**is UV-IWAS (i.e., massive-univariate analysis) without adjusting for the pleiotropic effect, with the Bonferroni correction applied.

Since we implemented our data analysis with summary statistics only, we converted each simulated data into necessary summary statistics. For binary traits, GWAS summary statistics were obtained under the true model (e.g., logistic regression), but the assumed stage 2 model was linear regression(Eq. (3)). Each simulation was based on 5,000 replicated datasets.

### 3.2 Simulation results

We first evaluated the empirical Type 1 error rates of the methods when the first imaging modality does not have a causal effect on the trait. We considered both 5% and 1% as the theoretical rates. The results shown inFigure 3imply that methods 1 and 2, which consider the pleiotropic effects, controlled Type 1 error rates well, in a slightly conservative manner. Method 2 was more conservative than our methods. It could be explained because each univariate analysis is already conservative (as discussed inKnutson et al. (2020))and the Bonferroni correction would make it more conservative. Methods 3 and 4 constantly produced inflated Type 1 errors as they do not adjust for pleiotropic effects in their models. Therefore, we only considered methods 1 and 2 in subsequent power evaluations.

**Fig. 3.**
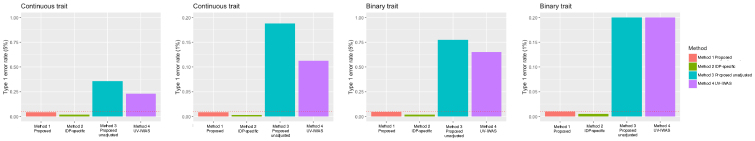
Type 1 error rates of different methods considered in this paper. The red horizontal line denotes the theoretical Type 1 error rates. The proposed method controls Type 1 error rate at the nominal rates of 5% and 1% in both continuous and binary traits. Methods 3 and 4 suffered from inflated Type 1 error rates as they do not consider pleiotropic effects. For visualizations, the y-axis of the plot was truncated at 0.2 when 1% Type 1 error is applied.

The summaries of power evaluations are provided inFigure 4. We considered Type 1 error rate of 5% when computing power in each scenario. The results were qualitatively similar for both continuous and binary traits. As expected, when all IDPs in the modality of interest have causal effects on the phenotype (“dense signal”), our method achieved higher power than Method 2, and the power increased as the degree of signal increased. Also, since we only used 10 IDPs from each modality, it is expected that the differences between the methods would be more prominent when signals are dense and the number of IDPs increases. When only one IDP had a causal effect on the phenotype, then the power between the two methods was similar. Although the latter result is unexpected because it is a setting that the minpmethod is expected to perform better, it might be explained by the strict Bonferroni correction applied in Method 2 (which was shown in the Type 1 error simulation).

**Fig. 4.**
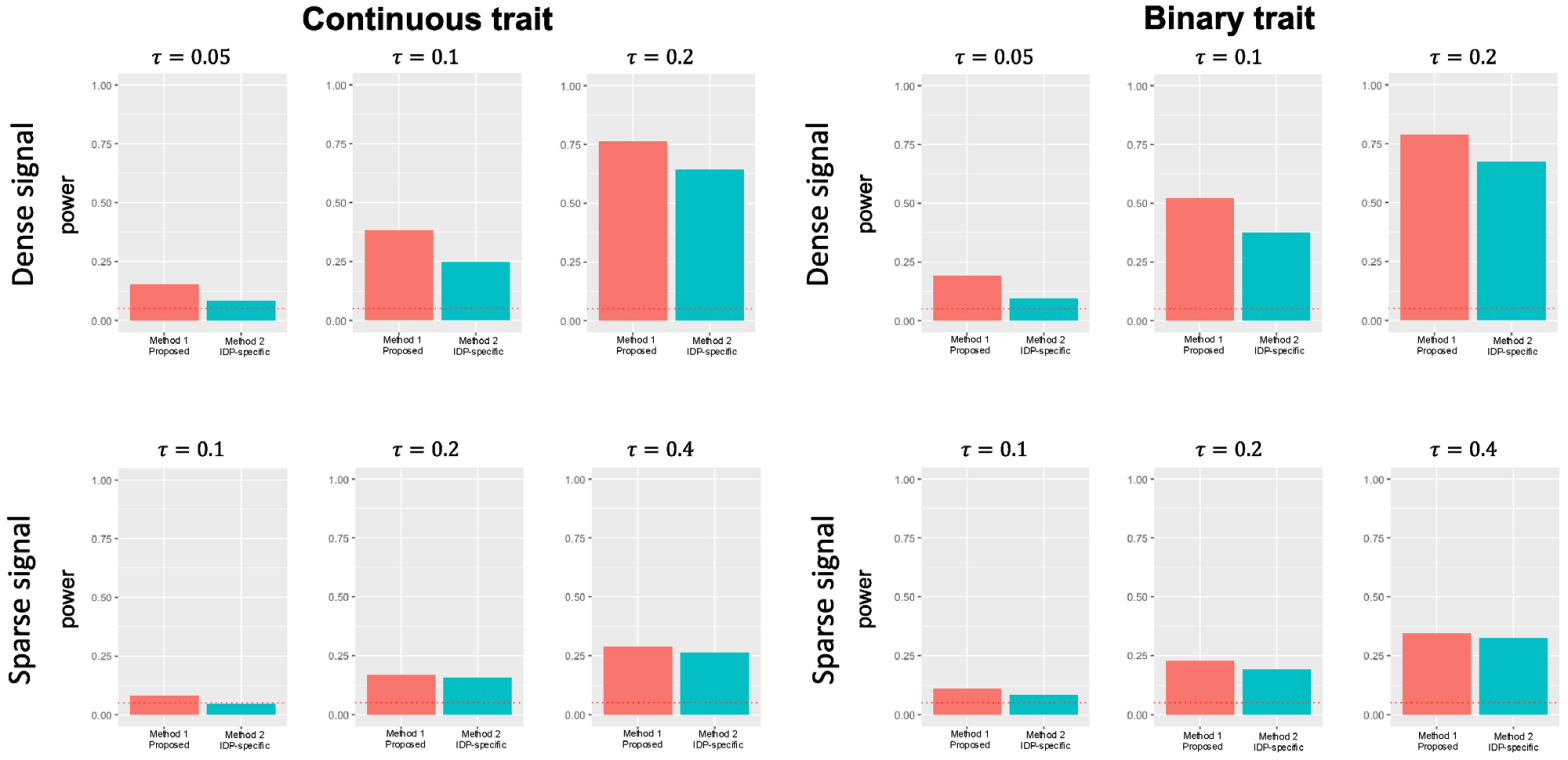
Summary of power analysis.$\tau^{2}$specifies the degree of signals where IDPs in the first modality cause the phenotype. “Dense signal” refers to the case where all IDPs in the first modality cause the phenotype, and “sparse signal” refers to the case where only one of the IDPs in the first modality is causal.

## 4 Data Analysis

### 4.1 Data availability

#### 4.1.1 UK Biobank: GWAS IDP summary statistics

We analyzed 1,436 T1-weighted sMRI features and 690 dMRI features (the IDs and descriptions of these features are available in the Github repository) and 210 resting-state functional connectivity metrics from the rfMRI full25×25correlation matrix (data field: 25750). As of 2020, UK Biobank collected approximately 40,000 subjects whose genotype and brain imaging data are both available. Among them, the GWAS summary statistics for each IDP (after adjusting for age, sex, 40 genetic principal components, scanning sites, head size, etc) is provided by Oxford Brain Imaging Genetics Server - BIG40 (https://open.win.ox.ac.uk/ukbiobank/big40/, led by Dr. Lloyd T. Elliott and Dr. Stephen Smith) (Elliott et al., 2018;Smith et al., 2021). The sMRI IDPs are categorized to regional and tissue volume (636 IDPs), cortical area (372 IDPs), cortical thickness (306 IDPs), cortical gray–white contrast (70 IDPs), regional and tissue intensity (41 IDPs). Also, the dMRI IDPs are categorized to WM tract FA (75 IDPs), WM tract MO (75 IDPs), WM tract diffusivity (300 IDPs), WM tract ICVF (75 IDPs), WM tract OD (75 IDPs), WM tract ISOVF (75 IDPs). Genotypes were imputed using the Haplotype Reference Consortium (HRC) as a reference panel (Bycroft et al., 2018).

We used the processing guidelines provided byKnutson et al. (2020)to choose SNPs to be used in our analysis. We used GWAS summary statistics on 2,310 heritable IDPs from 39,691 participants following (Smith et al., 2021), and used 1000G as reference genome for LD clumping. We employed a clumping radius of 1 M and set an$r^{2}$cutoff value of 0.5, as recommended byPrivé et al. (2019). This approach allowed us to remove loci with high linkage disequilibrium (LD) and keep the most significant SNP in each clump window based on the IDP GWAS results. When dealing with SNPs for a specific gene that are identified from different IDP GWAS clumps, we merged the SNP sets and treated them as a single SNP set for the gene. We refer tohttps://github.com/kathalexknuts/MVIWASfor more detailed procedures. Then, in our analysis, we only considered variants whose missing rate is less than 1%, and imputed all missing values with median values for each variant. FollowingGusev et al. (2016), we considered 1 Mb window around each gene to collect SNPs. Then, for each gene, we fitted a general linear model (GLM) to each preadjusted imaging feature to obtain weights. Following implications fromKnutson et al. (2020), we only considered gene–IDP pairs whosepvalues from theFtest are less than$5\times 10^{-5}$.

After applying LD clumping, the marginal GWAS vector for each gene was converted to regression coefficients for SNPs→IDP (i.e.,${\left({\hat{\alpha }}_{1k}^{\left(q\right)},\ldots ,{\hat{\alpha }}_{Jk}^{\left(q\right)}\right)}^{\prime }$) by first transforming the marginal (univariate) GWASzscore with$z/\sqrt{n-2+z^{2}}$(marginal regression coefficient) and multiplying it with the inverse ofRobtained from 1000 Genomes project.

#### 4.1.2 UK Biobank: GWAS AD summary statistics

In UK Biobank, various health statuses, including AD, have been phenotyped by ICD 10 codes (data field: 41270). The GWAS summary statistics for AD is provided by the Neale laboratory (https://www.nealelab.is/uk-biobank), where genotypes are imputed by HRC, UK10K, and 1000 Genomes. The summary statistics were obtained after adjusting for (inferred) sex, age, age^2^, sex×age, sex×age^2^, and 20 genetic principal components. Among 361,194 genotyped subjects, there were approximately 4,000 subjects diagnosed with AD.

#### 4.1.3 IGAP: GWAS AD summary statistics

International Genomics of Alzheimer’s Project (IGAP) (Lambert et al., 2013) is a large two-stage study based upon genome-wide association studies (GWAS) on individuals of European ancestry. In stage 1, IGAP used genotyped and imputed data on 7,055,881 SNPs to meta-analyze four previously published GWAS datasets consisting of 17,008 Alzheimer’s disease cases and 37,154 controls (The European Alzheimer’s disease Initiative – EADI the Alzheimer Disease Genetics Consortium – ADGC The Cohorts for Heart and Aging Research in Genomic Epidemiology consortium – CHARGE The Genetic and Environmental Risk in AD consortium – GERAD). In stage 2, 11,632 SNPs were genotyped and tested for association in an independent set of 8,572 Alzheimer’s disease cases and 11,312 controls. Finally, a meta-analysis was performed combining results from stages 1 and 2. GWAS summary statistics for gene–AD associations were obtained fromhttps://www.niagads.org/datasets/ng00036based on 17,008 AD cases and 37,154 healthy controls, after adjusting for sex, age, and (at least) 4 genetic principal components (Lambert et al., 2013).

### 4.2 Analysis results

#### 4.2.1 IGAP: instrumental genes and causal IDPs using IGAP GWAS AD summary statistics

As shown inFigure 5, the instrument genes identified by MV-Modality-IWAS were found on chromosomes 8 (4 genes, 9 genes) and 19 (39 genes, 38 genes) for sMRI and dMRI, respectively, but none was found in fMRI, at the Bonferroni-adjusted significance level of0.05. Among the genes on chromosome 8, 3 instrumental genes are shared between dMRI and sMRI, 37 instrumental genes are shared between dMRI and sMRI. No instrumental genes were identified for fMRI as expected, as the fMRI Independent Component Analysis (ICA) was performed at 25 dimensions, summarizing major patterns of brain activity. This approach of data summarizing may result in low (<5%) IDP heritability explained by genotype (Smith et al., 2021), which affects the Stage 1 model and subsequently then led to insignificant results in the Stage 2 testing.

**Fig. 5.**
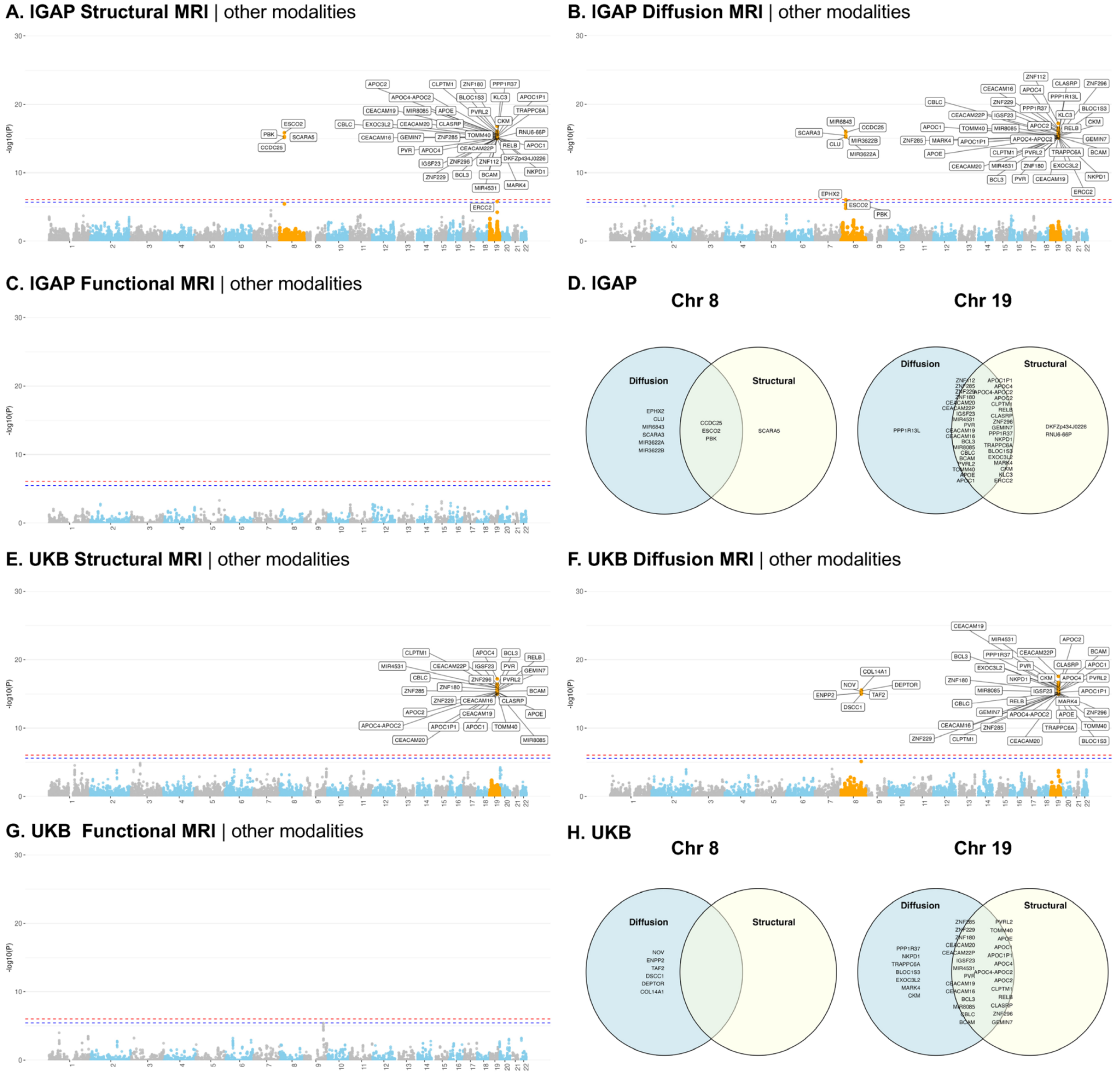
MV-Modality-IWAS analysis results using AD GWAS summary statistics from IGAP and UKB. Panels A–C showp-values for testing causality of each modality when adjusting for pleiotropic effects of other modalities and using each gene as instrumental variables. Panel D shows Venn diagram of the statistically significant genes identified in A–C, summarized by chromosomes 8 and 19. The analysis was performed at a Bonferroni-adjusted significance level, red dashed lines in panels A, B, and C represent the global significant threshold, that is,0.05​/​60,258; red dashed lines in panels A, B, and C represent the modality-specific significant threshold, that is,0.05​/​21,896,0.05​/​23,214,and0.05​/​15,148, respectively. Panels E–H show the results when using UK Biobank AD GWAS summary statistics. Still, UKB analysis was performed at a Bonferroni-adjusted significance level, red dashed lines in panels E, F, and G represent the global significant threshold0.05​/​54,082; blue dashed lines in panels E, F, and G represent the modality-specific significance level, that is,0.05​/​20,937,0.05​/​19,662,and0.05​/​13,483, respectively.

For dMRI, MV-Modality-IWAS identified 71 unique causal IDPs with instrumental genes on chromosomes 8 and 19, where 44 IDPs fall in the category of weighted-mean (WM) tract diffusivity, 6 IDPs were WM tract fractional anisotropy (FA), 15 IDPs falls in the category of WM tract intracellular volume fraction (ICVF), 3 IDPs fall in the category of WM tract isotropic or free water volume fraction (ISOVF), and 3 IDPs fall in the category of WM tract orientation dispersion index (OD).

For sMRI, MV-Modality-IWAS identified 135 unique causal IDPs with instrumental genes on chromosomes 8 and 19. Among these, 31 IDPs were cortical area, 4 IDPs were cortical gray–white contrasts, 54 IDPs were cortical thickness, 4 IDPs were regional and tissue intensities, and 42 IDPs were regional and tissue volume.

#### 4.2.2 UKB: instrumental genes and causal IDPs using UKB GWAS AD summary statistics

With UKB summary statistics, similar to using IGAP summary statistics, we found instrument genes on chromosomes 8 (0 gene, 6 genes) and 19 (27 genes, 34 genes) for sMRI and dMRIs, respectively. However, using UKB summary statistics, fewer instrumental genes were identified overall, particularly for sMRI where no instrumental genes were found on chromosome 8, compared with 4 genes identified using IGAP summary statistics. This discrepancy may be due to the median age difference between the datasets: the median age in UKB is 56.5 years, whereas IGAP considered late-onset AD cases with onset age>65 years, suggesting potentially stronger signals in IGAP with a larger number of cases used in generating its GWAS summary statistics. Additionally, it is also noteworthy that all instrumental genes identified on chromosome 19 for both dMRI and sMRI using UKB summary statistics are also identified using IGAP summary statistics. However, none of the 6 genes found by UKB for dMRI on chromosome 8 is identified using IGAP summary statistics.

With instrumental genes on chromosomes 8 and 19, MV-Modality-IWAS identified 71 unique causal dMRI IDPs for the UKB dataset. Among these 71 unique dMRI IDPs, 36 IDPs fall in the category of WM tract diffusivity, 2 IDPs in WM tract FA, 21 IDPs fall in the category of WM tract ICVF, 6 IDPs fall in the category of WM tract ISOVF, 1 IDP falls in the category of WM tract mean orientation (MO), and 5 IDPs fall in the category of WM tract OD. All causal IDP categories found in IGAP summary statistics were also identified with UKB, with the one additional category: WM tract MO. Additionally, a similar pattern was observed in both IGAP and UKB causal IDP results, where the majority of causal IDPs fell into the categories of WM tract diffusivity and WM tract ICVF.

With instrumental genes on chromosome 19, MV-Modality-IWAS identified 35 unique causal sMRI IDPs from the UKB dataset. Among these, 8 IDPs fall in the category of cortical area, 9 IDPs fall in the category of cortical thickness, 1 IDP falls in the category of regional and tissue intensity, and 17 IDPs fall in the category of regional and tissue volume. The causal IDP categories identified by UKB summary statistics are similar to those of IGAP, but with significantly fewer causal IDPs across all categories. Notably, no causal IDPs were found in cortical gray–white contrasts with UKB data.

Taken altogether, while IGAP and UKB GWAS summary statistics differ by many factors including preprocessing pipelines, the number of samples, the proportion of AD cases, age range, and others, V-Modality-IWAS identified genes on chromosomes 8 and 19 for sMRI and dMRI and no genes for fMRI in both datasets. These results show potential utility of the proposed method, warranting further investigations.

### 4.3 Comparisons

To test alternative methods of identifying causal IDPs, we compared our proposed approach with two additional strategies. With these comparisons, we aim to explore different underlying model assumptions and potential biases in identifying causal MRI–AD relationships.

#### 4.3.1 No adjustment for horizontal pleiotropic effects

In this comparison, we tested the same hypothesis without adjusting for pleiotropic effects from other modalities. The summaries ofpvalues are provided inFigure 6. In the sMRI analysis, ignoring pleiotropy led to the identification of nine additional genes on chromosome 2, but failed to detect the loci on chromosome 8 that were identified by MV-Modality-IWAS in the IGAP dataset. In UKB, however, no instrumental genes were detected, which yielded heterogeneity in results. In the dMRI analysis, ignoring pleiotropy led to the identification of 8 genes on chromosome 1 and 9 genes on chromosome 2, but failed to detect the loci on chromosome 8 that were identified by MV-Modality-IWAS in the IGAP dataset. In UKB, the loci on chromosomes 1 and 2 did not show any significance, but there was a loci (with 3 genes) on chromosome 22. In fMRI analysis, while UKB did not show any significant gene (which agreed with MV-Modality-IWAS results), IGAP detected 1 instrumental gene on chromosome 1 and 35 instrumental genes on chromosome 19. These noticeable differences in results indicate potential false positive genes from the lens of causal inference.

**Fig. 6.**
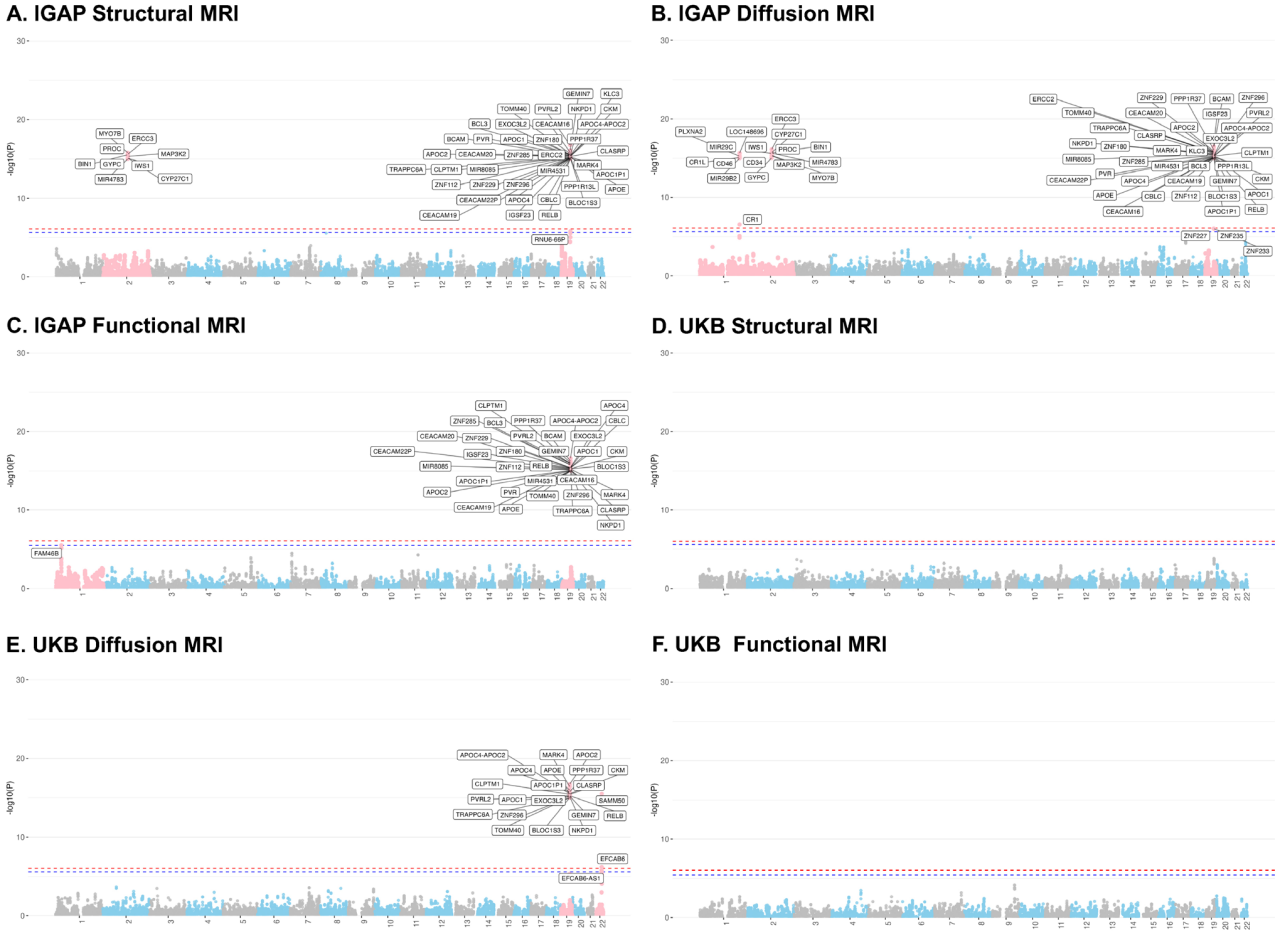
Analysis results using AD GWAS summary statistics without adjusting for other modalities. Panels A–C showp-values for testing causality of each modality when no pleiotropic effects are adjusted and using each gene as instrumental variables. The analysis was performed at a Bonferroni-adjusted significance level as described inFigure 5. Panels D–F show the results when using UK Biobank AD GWAS summary statistics and no pleiotropic effects are adjusted. UKB analysis was performed at a Bonferroni-adjusted significance level as described inFigure 5.

#### 4.3.2 MV-IWAS

In this comparison, we test each IDP individually while adjusting for all other IDPs within the same modality as well as IDPs from other modalities. To control for multiple comparisons in modality level testing, a Bonferroni correction was applied based on the number of tests conducted within each modality. Specifically, there were 699,007 tests in total being conducted in IGAP GWAS and 615,315 tests in total conducted in UKB GWAS. To keep MV-IWAS comparable with the proposed method, we applied MV-IWAS for each gene (rather than using all SNPs from 22 chromosomes). We did not find any statistical significance in both analyses. It is also important to note that our approach to performing MV-IWAS differs from that ofKnutson et al. (2020)in two ways: (1) in the original approach, all SNPs across all chromosomes were used in the Stage 1 model to impute each IDP, whereas we used only the SNPs within each gene being tested; (2) in the original MV-IWAS, only 14 IDPs were tested, so the Bonferroni correction had a much milder impact than in our case, where a substantially larger number of IDPs were tested individually.

These results support our proposal of testing MRI modalities as a whole unit. While testing each IDP individually offers better interpretation regarding causality in identifying causal IDPs, it is too restrictive that no statistically significant results were found using both IGAP and UKB data after applying Bonferroni correction. Analyzing each IDP individually necessitates a much stronger signal to achieve significant findings, which underscores the challenge of power loss with this approach.

## 5 Discussion

In this study, we were motivated by the large amount of MRI data available in the UK Biobank and the power loss associated with analyzing a large number of individual IDPs using existing methods such as MV-IWAS. To address this issue, we proposed an association test for the genetically regulated effects of a given MRI modality (sMRI, dMRI, or fMRI) on the progression of Alzheimer’s disease with high power. Our method views each MRI modality as a unitary entity and tests the genetically regulated effects of the modality as a whole. Finally, since each IDP contributes only a small proportion to the progression of AD, we propose a variance component test to test whether the overall genetically regulated effects of one modality contribute to the progression of AD while adjusting for the effects of other modalities as well as the residual genetic direct effects.

The proposed method, MV-Modality-IWAS, provides a new perspective on how to handle high-dimensional complex imaging data in genetic association studies. Our method significantly reduces the number of tests needed, resulting in higher power and a reduced need for family-wise error correction, especially when dealing with a large number of IDPs. Similar to MV-IWAS, our method adjusts for other genetically regulated effects and residual genetic direct effects, thus providing better control of Type I error than UV-IWAS. Furthermore, by collapsing the information in the modality being tested, fewer parameters are being tested, allowing our method to achieve higher power while controlling the Type I error rate. Overall, our method provides a powerful and efficient approach to analyzing complex imaging data in genetic association studies. However, there is a trade-off between power and causal interpretability. While the proposed method can identify the modality of the genetically regulated effect, we cannot pinpoint the specific IDPs through which the effect is mediated. Therefore, if the goal is to pinpoint specific IDPs that are causal for AD, a post-analysis can be conducted within the causal modality identified by MV-Modality-IWAS using MV-IWAS or other approaches, such as the Sum of Single Effects (SuSiE) method (Chan et al., 2024;Karageorgiou et al., 2023).

In general, causal inference on observational data is not without assumptions and limitations, and imaging genetic studies may also be subject to violations on assumptions. First, we only listed brain imaging data as potential exposures. In practice, there could be environmental/behavioral factors or other physiological measures (including -*omics*or other imaging types such as heart MRI (Zhao et al., 2023) and retinal optical coherence tomography (Zhao et al., 2024)) that could serve as potential exposures. In this case, it is expected that our causal argument would be weaker. Second, we considered horizontal pleiotropy in our settings only but not vertical pleiotropy, which might be the case in neuroimaging when a certain modality (e.g., sMRI) leads changes in other modality (e.g., fMRI). It is also important to note that the proposed method is primarily focused on statistical inferences rather than effect size estimation. If modality-level effect sizes are of interest, one may consider estimating modality-specific partial$R^{2}$, which provides a standardized measure of how much a linear approximation of variance in AD is uniquely explained by a specific modality. Despite these limitations, our proposed method makes one step closer in addressing high-dimensionality of pleiotropic effects in leveraging IDPs in studying genetic effects on AD. We also note that these are ongoing challenges in causal inference in genetics research where more research is needed to make valid inferences in such cases.

One possible extension is to use a dimension reduction method (e.g., principal component analysis or independent component analysis) to relax issues with multicollinearity when horizontal pleiotropy exists (Karageorgiou et al., 2023;Mo et al., 2022;Patel et al., 2024). It might require determination of dimensions in each modality. It could be a popular method such as principal component analysis or independent component analysis, or more flexible methods that decompose and factorize multimodal data into “shared and individual” variations (Lock et al., 2013,^2022^;Park & Lock, 2020). Although it is not evaluated in this paper because it requires access to individual-level genotype and imaging data, as well as computational costs to train machine learning models for each gene–IDP pair, we believe such approaches would greatly enhance the scope of our method. Especially if there is availability of dimensionality-reduced composite score GWAS summary statistics, and with clear evidence that the genetic variation is captured by the composite scores, the dimensionality-reduced composite scores can be used to replace the IDPs in the analysis. Another potential extension of the proposed method is to the flexibility on IDP grouping scheme. In real data applications, grouping by modality is not required by the modeling approach, and alternative grouping schemes may be considered—such as organizing IDPs by feature type (e.g., surface area, volume, thickness). However, when doing so, it is important to carefully consider the impact of multicollinearity and multiple testing when selecting the groups.

## Data Availability

The GWAS summary statistics data used in this study are publicly available, with details provided inSection 4.1. Codes used in simulation studies and data analyses are available athttps://github.com/junjypark/MV_Modality_IWAS.
